# Genetic Diversity and Gene Flow of the Ectomycorrhizal Mushroom *Lactarius hatsudake* in Southern China: Evidence from SSR Markers

**DOI:** 10.3390/jof12040280

**Published:** 2026-04-15

**Authors:** Shatong Yang, Mingwei Mao, Jieyu Huang, Bing Gu, Kuan Zhao

**Affiliations:** Key Laboratory of Natural Microbial Medicine Research of Jiangxi Province, College of Life Science, Jiangxi Science and Technology Normal University, Nanchang 330013, China; yshatong@163.com (S.Y.); mmw0403@163.com (M.M.); jy_huang2002@163.com (J.H.)

**Keywords:** milk cap mushroom, SSR, population structure, genetic diversity, genetic variation

## Abstract

*Lactarius hatsudake* is an ecologically and economically significant wild edible mushroom in southern China. To elucidate its population genetic diversity, differentiation, and evolutionary history, we analyzed 172 fruiting bodies from eight geographic populations (AQ, BS, DZ, JS, NC, PT, SG, YX) across seven provinces in the western and eastern regions of southern China using five highly polymorphic simple sequence repeat (SSR) markers. Combined with STRUCTURE clustering, discriminant analysis of principal components (DAPC), unweighted pair group method with arithmetic mean (UPGMA), and analysis of molecular variance (AMOVA), the results revealed high polymorphism across the studied loci (mean PIC = 0.842). A total of 75 alleles were identified, averaging 15 alleles per locus. At the population level, the mean effective number of alleles (Ne) was 4.023, and the mean unbiased gene diversity (uH) was 0.768. The NC population exhibited the highest genetic diversity (uH = 0.796), whereas the BS population showed relatively lower diversity (uH = 0.647). Clustering analyses (STRUCTURE, DAPC, and UPGMA) consistently identified two distinct genetic clusters (K = 2). Cluster I consisted of populations AQ, PT, BS, and SG, while Cluster II was composed of the remaining four populations. Notably, individuals from AQ and NC displayed significant genetic admixture, suggesting a transitional zone. AMOVA revealed that the majority of genetic variation (83%) resided within populations and 17% among populations. Moderate population differentiation (ENA-corrected global Fst = 0.102) and admixture signals suggest non-negligible connectivity among populations.

## 1. Introductiona

*Lactarius hatsudake* is an economically and ecologically significant wild mushroom belonging to the family Russulaceae [1,2]. It is widely distributed across southern Asia and associated with pine trees [3]. It is rich in phenolic compounds and structurally diverse polysaccharides, such as glucans and galactans, which endow the mushroom with significant antitumor and antioxidant potential [4,5,6].

Relatively few studies have examined the genetic structure of its natural populations. To the best of our knowledge, based on systematic literature, two representative studies have investigated the genetic diversity and population structure of this milk cap mushroom. An early population genetic analysis used an ITS fragment (612 bp, 31 SNPs) to examine 41 individuals from four natural populations in Hunan, Guizhou, Guangxi and Yunnan Provinces in southern China [7]. This study revealed high genetic diversity and genetic differentiation among populations, with 18 haplotypes identified and evidence of gene flow, although no significant correlation between genetic differentiation and geographical distance was detected. More recently, our team developed the first set of five genome-derived SSR primers for this species and applied them to 102 samples belonging to eight populations from three provinces in southwestern China [8]. The study revealed high levels of genetic differentiation (AMOVA indicated 60% variation among populations) and significant inbreeding effects (widespread heterozygote deficiency). However, as the above two studies are limited to the sampling areas, the conclusions are tentative. Furthermore, does this species exhibit a phylogeographic structure shaped by historical processes across larger spatiotemporal scales?

Microsatellite or simple sequence repeat (SSR) markers derived from whole-genome sequences are highly effective for population genetic studies due to their codominant inheritance, high mutation rates, and abundant polymorphism [9,10,11]. They are particularly well-suited for investigating microevolutionary processes, population structure, and gene flow in non-model organisms [12,13,14]. For instance, SSR markers have been extensively applied to assess genetic diversity and differentiation in the cultivated button mushroom *Agaricus bisporus*, revealing a narrow genetic base in most cultivated lines [15]. These findings have provided key information for breeding and conservation programs. In the jelly mushroom *Auricularia heimuer*, 30 SSR markers have been used to survey 72 wild strains from major distribution areas in China, North Korea and Russia, revealing high allelic richness and pronounced genetic differentiation across its range [16]. A subset of highly informative loci can uniquely fingerprint all strains, providing an efficient tool for germplasm identification, conservation and targeted breeding. Similarly, SSR-based analyses of *Lentinula edodes* detected clear genetic differentiation between wild and cultivated groups and among geographically distinct wild populations, highlighting the roles of domestication and biogeographic barriers in shaping genetic structure [17].

The primary objective of this study is to characterize the genetic diversity, population structure and differentiation of *L. hatsudake* using highly polymorphic SSR markers. By integrating geographical distribution data, we further examined the influence of geographical barriers and anthropogenic factors on its current genetic patterns. This research contributes a multi-faceted analysis of the population genetics of *L. hatsudake* in southern China, providing a scientific basis for the conservation and sustainable utilization of its genetic resources, as well as insights into its evolutionary history.

## 2. Materials and Methods

### 2.1. Sample Collection, Identification and DNA Extraction

**Figure 1:**
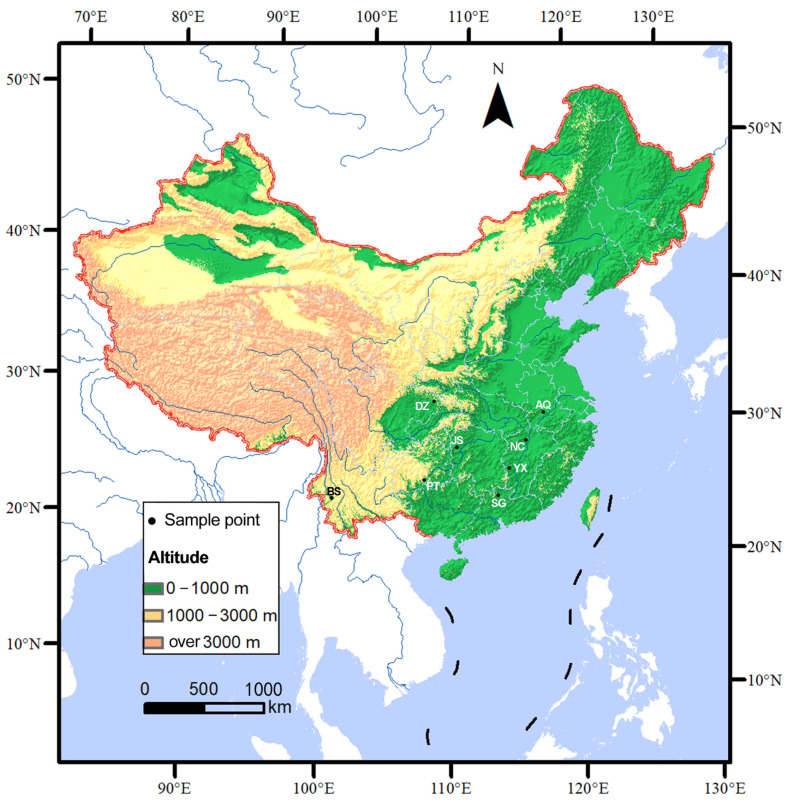
The sampling sites are represented by black dots, and details of their abbreviated codes are shown in Table 1.

From the year 2023 to 2025, sample collection was conducted across southern China, spanning an east–west distance of approximately 2000 km. A total of 172 fruiting bodies were collected from eight representative geographic populations, namely Baoshan, Dazhou, Jishou, Pingtang, Anqing, Nanchang, Shaoguan, and Yongxin. The geographic locations of the eight sites are shown in Figure 1 (map generated with ArcGIS v10.0, https://www.esri.com/en-us/arcgis/products/arcgis-for-personal-use/overview, accessed on 8 February 2026). All sampling sites were selected based on the natural distribution range of *L. hatsudake*, local seasonal fruiting patterns, and wild mushroom harvesting traditions, ensuring the representativeness of the eight geographic populations, including the BS population from the western margin of the species’ distribution in southern China. Within each site, the fruiting bodies were collected from the natural habitats according to the collection methods introduced in the previous literature and supplemented by local mushroom markets. After careful confirmation of species identity through morphological and microscopic examination, specimens were promptly dried and preserved in silica gel [18]. Detailed information for each population, including geographic coordinates, elevation, and main associated host plants, is provided in Table 1.

The genomic DNA was isolated following a modified CTAB protocol [19]. DNA integrity was assessed via 1% agarose gel electrophoresis. DNA samples exhibiting clear bands, high purity, and structural integrity were diluted to a final concentration of 50 ng/µL and stored at −20 °C for subsequent analysis.

### 2.2. SSR Marker Screening and Genotyping Analysis

Five highly polymorphic SSR markers (Lh1, Lh4, Lh5, Lh8, Lh14), previously developed by Zhao et al., were utilized in this study [8]. PCR amplifications were performed in a 25 μL reaction volume containing 1 μL of template DNA (50 ng/μL), 1 μL each of forward and reverse primers (5 μmol/L), 10.5 μL of 2 × Taq Master Mix (Sangon Biotech, Shanghai, China), and 10.5 μL of ddH_2_O. PCR was performed at 95 °C for 3 min, 35 cycles of 95 °C for 30 s, 54 °C for 30 s, 72 °C for 30 s, and a final extension at 72 °C for 10 min. PCR products were separated via capillary electrophoresis (Sangon, Shanghai, China), and fragment sizes were sized using GeneMarker v2.9.0 to determine the genotype of each sample at every locus [20]. To evaluate marker polymorphism, the polymorphism information content (PIC) for each SSR locus was calculated using Cervus 3.0 [21].

### 2.3. Data Analysis

To systematically elucidate the population genetic characteristics of *L. hatsudake* in southern China, a multidimensional analysis based on SSR genotyping data was performed. GenAlEx 6.5 was utilized to calculate fundamental genetic diversity parameters for each population, including the number of alleles (Na), effective number of alleles (Ne) and unbiased gene diversity (uH) [22,23]. To investigate mating systems and reproductive dynamics, tests for deviation from the Hardy–Weinberg equilibrium (HWE) were conducted using the same software. Observed heterozygosity (Ho) was compared with expected heterozygosity (He) at each locus to detect heterozygote deficiency or excess, providing insights into reproductive strategies. Additionally, linkage disequilibrium (LD) between loci was assessed using Genepop v4.7.0 to evaluate genetic recombination levels within populations [24].

Null allele frequencies were estimated for each locus and population using the EM algorithm implemented in Genepop v4.7.0 [24]. To correct for the potential bias introduced by null alleles, pairwise fixation index (Fst) values were recalculated using the ENA (excluding null alleles) method in FreeNA. Based on the 28 corrected pairwise Fst values, the 95% confidence interval for the global mean Fst was estimated by bootstrap resampling with 1000 replicates using the same software [25,26,27,28].

Population genetic structure and differentiation were analyzed using analysis of molecular variance (AMOVA) implemented in GenAlEx 6.5 to partition total genetic variation into two components: among populations and within populations [29]. Pairwise genetic differentiation was also quantified using Rst, which is based on the stepwise mutation model (SMM) for microsatellites [30].

To account for differences in sample size among populations, allelic richness (Ar) was calculated using the rarefaction method implemented in FSTAT v2.9.3 [31,32]. The rarefaction procedure standardizes allelic counts to the smallest sample size (*n* = 20) across all populations, allowing unbiased comparison of genetic diversity [33].

To test for isolation-by-distance (IBD), Mantel tests were performed using the vegan package in R (https://cran.r-project.org/web/packages/vegan/, accessed on 15 February 2026) with 9999 permutations. Pairwise linearized genetic distances (Fst/(1 − Fst)) based on ENA-corrected Fst values were correlated with log-transformed geographic distances (km) calculated from GPS coordinates (Table 1). Pearson’s product-moment correlation was used as the test statistic. This approach follows the classic Mantel test framework and is widely used in population genetics to assess spatial patterns of genetic divergence [34,35]. The use of log-transformed geographic distances follows the theoretical expectation for IBD in two-dimensional habitats [36].

To visualize and further resolve population structure, three complementary clustering approaches were applied. First, Bayesian clustering was performed using STRUCTURE 2.3.4, with the number of clusters (K) ranging from 1 to 8 [37,38,39]. Five independent runs were executed for each K, consisting of 1,200,000 Markov Chain Monte Carlo (MCMC) iterations with a burn-in period of 200,000 generations [37]. The optimal K was determined using the ΔK method. Second, Discriminant Analysis of Principal Components (DAPC) was conducted using the adegenet package (http://adegenet.r-forge.r-project.org/, accessed through 19 January 2026) in R. Cross-validation was employed to determine the optimal number of retained principal components (PCs) to maximize the efficacy of the discriminant analysis while avoiding overfitting [40]. Finally, an unweighted pair group method with arithmetic mean (UPGMA) phylogenetic tree was constructed in MEGA X based on Nei’s unbiased genetic distance [41]. The reliability of the topology was assessed using 1000 bootstrap replicates, and the resulting consensus tree was visualized using the iTOL (https://itol.embl.de/, accessed on 19 January 2026) online platform [42].

## 3. Results

### 3.1. Polymorphism of SSR Markers and Population Genetic Diversity

Genetic analysis of 172 *L. hatsudake* samples from eight geographic populations identified a total of 75 alleles across the five SSR loci (Table S1). The number of observed alleles (Na) per locus ranged from 12 (Lh1) to 21 (Lh5). Observed heterozygosity (Ho) varied from 0.006 (Lh1) to 0.535 (Lh14), whereas expected heterozygosity (He) ranged from 0.803 (Lh8) to 0.865 (Lh14). All five SSR markers proved to be highly informative, with PIC values ranging from 0.803 to 0.865 (Table 2).

Geographical variation in genetic diversity indices was observed at the population level (Table 3). Allelic richness (Ar), standardized to a sample size of 20 individuals, ranged from 5.400 (SG) to 7.983 (NC). The effective number of alleles (Ne) ranged from 2.990 (AQ) to 5.092 (NC), averaging 4.023 across populations. Unbiased gene diversity (uH) was highest in NC (0.796) and lowest in BS (0.647), with an overall average of 0.768.

### 3.2. Hardy–Weinberg Equilibrium and Linkage Disequilibrium

**Figure 2:**
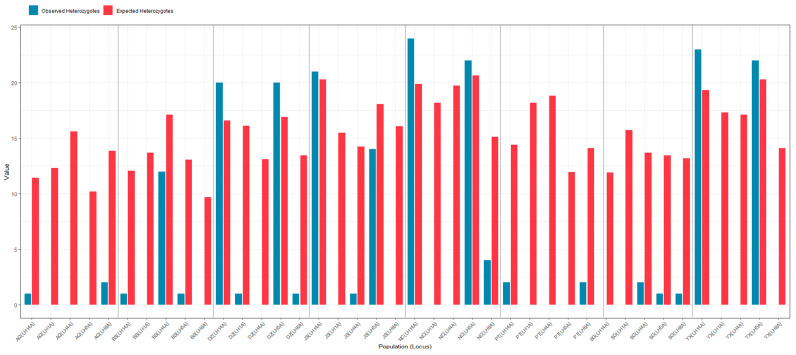
Chi-square tests for Hardy–Weinberg equilibrium within eight local populations of *Lactarius hatsudake* in southern China.

Hardy–Weinberg equilibrium tests revealed widespread and highly significant deviations (*p* < 0.001) at all five SSR loci across the eight *L. hatsudake* populations (Figure 2). Across all loci, the values of Ho were consistently lower than those of He. Significant deviations from HWE (*p* < 0.05) were detected across all markers. For instance, the value of Ho was zero or extremely low across all loci in the AQ population, with similar patterns of severe deficiency observed in the PT and SG populations.

In contrast to the widespread deficiency, certain populations exhibited heterozygosity levels approaching or exceeding expectations at specific loci. For example, in the DZ population, the observed number of heterozygotes at loci LH5 and LH14 (20 and 20, respectively) closely approached the expected numbers (16.9 and 16.6).

Linkage disequilibrium tests across populations (Fisher’s method in Genpop) indicated that most locus pairs were not in significant LD. After Bonferroni correction for 10 pairwise comparisons (α = 0.005), only Lh1–Lh14 remained significant (*p* = 0.0036), whereas all other locus pairs were non-significant (Table S3).

Null allele analysis using the EM algorithm revealed that null allele frequencies ranged from 0 to 0.461 across most locus–population combinations (Table S2). For instance, LH1 exhibited null allele frequencies between 0.396 and 0.453 across all eight populations, while LH4 ranged from 0.136 to 0.486. Notably, some populations (DZ, NC, YX) showed null allele frequencies of zero at loci LH5 and LH14, suggesting locus-specific variation in null allele occurrence.

### 3.3. Population Genetic Differentiation and Gene Flow

Analysis of Molecular Variance (AMOVA) revealed that the majority of genetic variation (83%) in southern Chinese *L. hatsudake* populations resides within populations, while variation among populations accounts for only 17% (Table 4). The among-population component was statistically significant, indicating significant genetic differentiation among the eight *L. hatsudake* populations.

Pairwise Fst values were corrected for null alleles using the ENA method in FreeNA. The global mean Fst after correction was 0.103, indicating moderate genetic differentiation among populations. For comparison, the uncorrected global mean Fst was 0.149. Pairwise corrected Fst values are provided in Table S4.

Pairwise comparisons of genetic differentiation (Rst) revealed distinct geographic patterns (Table S5). Several comparisons involving BS, DZ, JS, and PT show relatively high differentiation, for example, between the BS and JS populations (Rst = 0.255), between BS and DZ (Rst = 0.243), and between PT and YX (Rst = 0.248), suggesting limited gene flow among these population pairs. In contrast, many comparisons, particularly those among AQ, NC, SG, and YX, fall within an intermediate range (Rst = 0.142–0.215), which is consistent with comparatively stronger genetic connectivity. Notably, an exceptionally low Rst value (Rst = 0.018) is detected between the geographically distant BS and SG populations, and similarly low values are observed between BS and PT (Rst = 0.048) and between DZ and YX (Rst = 0.060), indicating unexpectedly high genetic similarity despite considerable geographic separation. Conversely, the strongest genetic differentiation (Rst > 0.24) is found between the BS and JS populations (Rst = 0.255), between PT and YX (Rst = 0.248), and between BS and YX (Rst = 0.241).

Mantel tests revealed no significant correlation between linearized genetic distances (Fst/(1 − Fst)) and log-transformed geographic distances (Pearson’s r = −0.039, *p* = 0.538), indicating that contemporary geographic distance does not explain the observed genetic structure (Figure S1).

### 3.4. Population Structure

**Figure 3:**
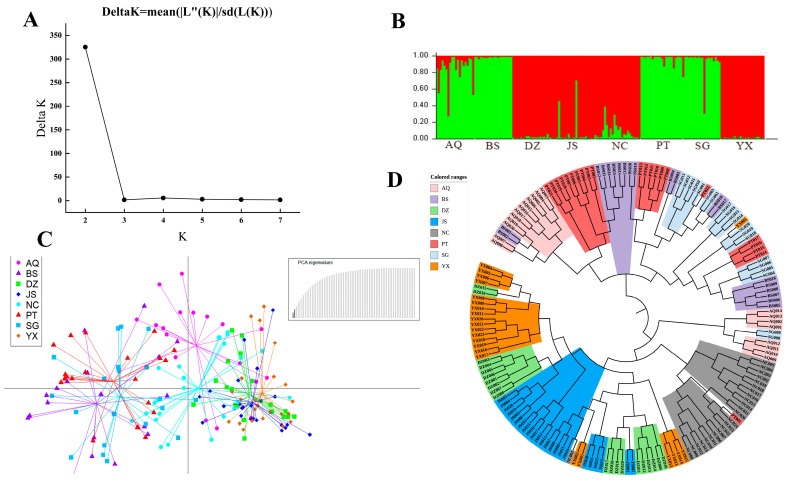
Genetic clusters of *Lactarius hatsudake* samples and their geographic distributions. (**A**) Delta K was calculated to estimate the optimal genetic population number K. (**B**) STRUCTURE ver.2.3.4 was used to assign each of the 172 samples to a genetic subpopulation and visualize the probability of belonging to each subpopulation (K = 2). Samples are arranged in a row and grouped by geographic subpopulation within the whole sample. Each color represents the isolate belonging to a different genetic subpopulation. (**C**) Genetic clustering using discriminant analysis of principal components (DAPC) of 172 samples from eight *L. hatsudake* geographic populations. (**D**) UPGMA dendrogram of eight geographic populations of *L. hatsudake* based on Nei’s genetic distance at five SSR loci.

In the STRUCTURE analysis, the ΔK statistic peaked at K = 2 (Figure 3A), Genetic clustering grouped the populations into two major clusters: AQ, PT, BS, and SG formed Cluster I, whereas JS, NC, DZ, and YX were assigned to Cluster II. Notably, varying degrees of genetic admixture were observed, particularly in individuals from the populations of AQ, SG, and YX.

The DAPC results corroborated the STRUCTURE analysis (Figure 3C), revealing clear boundaries between the two genetic groups. The two clusters exhibited distinct separation in the multivariate space. Meanwhile, individuals with admixed ancestry—primarily from the populations of AQ and NC—were positioned in the intermediate region between the main clusters.

Furthermore, the UPGMA phylogenetic tree (Figure 3D) resolved the eight populations into two major clades, fully corresponding to the genetic clusters identified by STRUCTURE and DAPC. The populations of AQ, BS, PT, and SG formed one clade, while the remaining four populations formed the second, with high bootstrap support at the branch nodes.

## 4. Discussion

This study provides a multi-faceted analysis of the genetic diversity, structure, and differentiation of *Lactarius hatsudake* populations across southern China using five SSR molecular markers. The five SSR loci showed a high mean PIC of 0.842, indicating that the markers were highly informative for detecting micro-evolutionary variation. At the population level, *L. hatsudake* exhibited elevated genetic diversity, with a mean unbiased gene diversity (uH) of 0.768 and a mean effective number of alleles (Ne) of 4.023. These values are comparable to those previously reported for *L. hatsudake* in Southwest China (uH = 0.674–0.809) and are generally higher than estimates for other higher fungi, such as *Tricholoma matsutake* (mean He = 0.67), *Boletus edulis* (mean He = 0.62) and *Lentinula edodes* (mean He = 0.76) [8,17,43,44]. The observed high genetic variation may be attributed to the species’ extensive geographic range, large effective population sizes, and its ECM lifestyle. Additionally, stable host pine habitats (*Pinus massoniana*) likely play a key role in buffering populations from environmental fluctuations and preventing diversity loss.

Despite this overall pattern, genetic diversity varied markedly among populations. After standardizing the sample size using Ar, the diversity rankings remained highly consistent with those based on uH (Table 3). The NC population still showed the highest Ar (7.98), while BS and SG showed the lowest (5.60 and 5.40, respectively). This confirms that the observed diversity differences are biologically meaningful and not merely sampling artifacts.

Notably, significant deviations from HWE were detected at the majority of loci across all populations, with Ho consistently lower than He, indicating a widespread heterozygote deficiency. Such heterozygote deficiency is a common phenomenon in fungal population genetics. It has been documented in species with inherent selfing mechanisms, such as the secondary homothallic *Agaricus bisporus* as well as in obligately outcrossing species prone to ecological inbreeding, like *Morchella* spp. and *Suillus* spp., where limited spore dispersal and clonal growth often lead to mating among closely related individuals [15,45,46]. Although *L. hatsudake* is presumed to be heterothallic, inbreeding is prevalent in its natural populations. This finding is consistent with a previous study [8].

In addition to these biological factors, technical artifacts may also contribute to the observed heterozygote deficiency. Null allele analysis revealed that null allele frequencies ranged from 0 to 0.461 across most locus–population combinations (Table S2), indicating that null alleles are common in our dataset [25]. However, as demonstrated by Chapuis & Estoup, null alleles lead to overestimation of Fst values but do not fundamentally alter the relative patterns of population differentiation when multiple independent loci are used [47]. Therefore, while null alleles may partially explain the low Ho, the core findings of this study (K = 2 structure and moderate differentiation) remain robust, as they are supported by methods resilient to this artifact (ENA-corrected Fst, DAPC, UPGMA).

The combined results from STRUCTURE, DAPC, and UPGMA consistently partitioned the southern Chinese *L. hatsudake* populations into two distinct genetic clusters (K = 2), which coincides with previous findings in Southwest China (K = 2) [8]. In southwestern China, although the geographical distribution of the milk cap mushroom was topographically fragmented in the Hengduan Mountains, the genetic structure is not a reflection of contemporary geographic isolation, as the genetic variation was not found to correlate with geographic distance. Similarly, in southern China, this study also observed a geographically mosaic pattern that mirrors this decoupling of genetic and geographic distance. For example, the BS and PT populations cluster together with AQ and SG, whereas DZ groups with YX and NC despite their geographic separation.

This decoupling of genetic and geographic distance suggests that contemporary geographic divisions alone cannot explain the observed genetic structure. Instead, the pattern points to the influence of historical factors, most likely associated with Quaternary glacial oscillations. During glacial periods, populations may have retreated into multiple isolated refugia, where genetic divergence accumulated, followed by postglacial range expansions and secondary contacts that reshuffled genetic composition across space [48]. This interpretation aligns with Quaternary biogeographic theory and the well-documented refugia dynamics in mainland China [49,50]. Notably, host tree species of *L. hatsudake*, including *Pinus massoniana* and *P. koraiensis*, exhibit clear genetic divisions corresponding to putative northern and southern refugia [51,52]. We hypothesize that *L. hatsudake* may have persisted in multiple, geographically separated refugia within its present range in southern China during the Last Glacial Maximum (LGM), although the precise locations of these refugia remain to be determined [53]. The limited number of SSR markers and the lack of mutation rate calibration preclude confident dating of divergence events to the Pleistocene. Therefore, the LGM double-refugia scenario should be considered a preliminary hypothesis. As climates warmed in the postglacial period, lineages from these distinct refugia likely expanded outward, resulting in the intermingled lineage distribution observed today. While this theoretical model offers a plausible explanation for the current genetic pattern, further validation through additional evidence and more detailed investigations is needed.

While our previous study focused on southwestern China, the present study extends these findings across a much broader landscape. AMOVA showed that 17% of genetic variation resided among *L. hatsudake* populations in southern China, whereas in Southwest China, up to 60% was among populations [8]. The reason for this pronounced discrepancy (17% vs. 60%) likely lies in differences in regional topography and habitat connectivity. The Southwest region is characterized by the Hengduan Mountains, where alpine gorges and deep valleys act as formidable barriers to gene flow [54,55]. In contrast, the current study area extends into Southeastern China, a region dominated by low mountains and rolling hills. The relatively gentle topography and the continuous distribution of host forests (*Pinus massoniana*) in the southeast significantly facilitate long-distance spore dispersal and gene exchange, thereby effectively reducing population differentiation (mean corrected Fst = 0.102) [56,57].

Rst further corroborates this landscape genetics pattern. Several comparisons involving the BS, DZ, JS and PT populations show moderate to high differentiation (in many cases Rst > 0.20), retaining the signature of isolation imposed by rugged montane terrain, where geographic barriers promote genetic drift [55]. By contrast, comparisons among AQ, NC, SG and YX, and between these populations and nearby sites, generally yield lower and more homogeneous Rst values, indicating comparatively stronger genetic connectivity. In the areas where these latter populations occur, more continuous pine-forest habitats and the absence of major topographic barriers are likely to have sustained higher levels of historical and contemporary gene flow, thereby slowing the accumulation of population divergence [58]. The extremely low Rst between BS and SG, together with similarly low values between BS and PT and between DZ and YX, indicates unexpectedly high genetic similarity between populations that are geographically distant and separated by substantial topographic relief. These anomalies are unlikely to be explained solely by short-range stepwise dispersal and suggest occasional long-distance gene flow, potentially mediated by strong wind events, animal vectors, or human-assisted movement of mycorrhizal seedlings and fruiting bodies [59,60].

The Mantel test further supported the lack of a simple isolation-by-distance pattern (Pearson’s r = −0.039, *p* = 0.538). Similar results have been reported in a previous study on *L. hatsudake* from South China (*p* = 0.32), as well as in its pine host *Pinus koraiensis* from Northeast Asia [7,61]. This result is consistent with the decoupling of genetic and geographic distances observed in pairwise Rst comparisons (low differentiation between distant BS and SG) and aligns with the proposed LGM double-refugia scenario. The absence of significant IBD suggests that contemporary geographic distance is not a major driver of genetic differentiation in *L. hatsudake*; instead, historical vicariance and subsequent range expansions have left a stronger imprint on the current population structure.

Although Mantel tests revealed no significant isolation-by-distance, the relatively low number of SSR markers may limit the power to detect fine-scale spatial patterns. Future studies with higher-density genomic markers and additional sampling sites are needed to further disentangle the roles of historical vicariance and contemporary landscape features in shaping the genetic structure of *L. hatsudake*.

## 5. Conclusions

This study revealed high levels of genetic diversity and gene flow among populations of the ectomycorrhizal mushroom *Lactarius hatsudake* in southern China. The observed high diversity provides a foundation for the species’ adaptive potential and informs conservation strategies for this edible fungal resource. Widespread heterozygote deficiency was detected across populations, suggesting that inbreeding or fine-scale genetic structuring may play a role in local population dynamics, with null alleles also contributing to the observed pattern. Population structure analyses identified two distinct evolutionary lineages, whose distribution does not correspond simply to contemporary geographic distance. Moderate genetic differentiation (global corrected Fst = 0.102) was observed among populations, suggesting potential connectivity. The lower differentiation observed in the eastern part of the range, as exemplified by the populations AQ, NC, SG, and YX, may reflect more continuous host pine forests and gentle topography, which could facilitate spore-mediated gene exchange. Collectively, these results demonstrate that gene flow occurs across southern China, yet its pattern is not simply governed by geographic distance.

## Figures and Tables

**Table 1:** Information on *Lactarius hatsudake* sample collection sites in southern China.

Code	City	Province	Sample Size	Altitude (Meters Above Sea Level)	Latitude	Longitude	Main Host Plant
AQ	Anqing	Anhui	20	400–800 m	30°50′59″ N	116°21′35″ E	*Pinus massoniana*
BS	Baoshan	Yunnan	20	1200–1600 m	24°49′40″ N	99°36′18″ E	*Pinus massoniana*
DZ	Dazhou	Sichuan	20	800–1200 m	31°8′45″ N	107°42′27″ E	*Pinus massoniana*
JS	Jishou	Hu’nan	23	400–600 m	28°16′27″N	109°40′39″E	*Pinus massoniana*
NC	Nanchang	Jiangxi	24	50–200 m	28°45′30″ N	115°44′5″ E	*Pinus massoniana*
PT	Pingtang	Guizhou	22	600–1500 m	25°49′21″ N	107°19′22″ E	*Pinus massoniana*
SG	Shaoguan	Guangdong	20	600–1000 m	24°41′13″ N	113°36′19″ E	*Pinus massoniana*
YX	Yongxin	Jiangxi	23	300–500 m	26°56′43″ N	114°14′33″ E	*Pinus massoniana*
Total			172				

**Table 2:** Polymorphism parameters of five SSR markers.

ID	Na	Ho	He	PIC
Lh1	12	0.006	0.849	0.828
Lh4	15	0.087	0.860	0.860
Lh5	21	0.483	0.857	0.857
Lh8	13	0.058	0.803	0.803
Lh14	17	0.535	0.865	0.865
Mean	15.6	0.234	0.860	0.842

**Table 3:** Mean allelic richness and allelic diversity (±standard error) at the five SSR loci within eight local populations of *Lactarius hatsudake* in southern China.

Population	Ar	Ne	uH
AQ	5.600 ± 0.800	2.990 ± 0.440	0.655 ± 0.048
BS	5.600 ± 2.417	3.497 ± 0.889	0.647 ± 0.062
DZ	7.400 ± 2.577	4.669 ± 0.721	0.781 ± 0.041
JS	7.051 ± 2.851	4.441 ± 1.078	0.748 ± 0.047
NC	7.983 ± 2.658	5.092 ± 0.769	0.796 ± 0.042
PT	6.333 ± 2.269	4.108 ± 0.936	0.720 ± 0.061
SG	5.400 ± 0.800	3.258 ± 0.368	0.697 ± 0.031
YX	7.466 ± 3.164	5.003 ± 1.032	0.782 ± 0.047
Mean	6.400 ± 0.842	4.023 ± 0.758	0.768 ± 0.043

Ar = allelic richness (standardized to a sample size of 20 individuals using the rarefaction method); Ne = effective number of alleles; uH = unbiased allelic diversity.

**Table 4:** Two-level hierarchical AMOVA of *Lactarius hatsudake* in southern China.

Source	df	SS	MS	Est. Var.	%Contribution
Among Populations	7	241.373	34.482	1.312	17 ***
Within Populations	164	1032.500	6.296	6.296	83 ***
Total	171	1273.872		7.608	100

SS: sum of squared values; MS: mean squared values; ***, *p* < 0.001.

## Data Availability

The original contributions presented in this study are included in the article/Supplementary Material. Further inquiries can be directed to the corresponding authors.

## Supplementary Materials

The following supporting information can be downloaded at: https://www.mdpi.com/article/10.3390/jof12040280/s1, Table S1: Multilocus genotypes of *Lactarius hatsudake* from southern China. Table S2: Null allele frequencies for each locus and population. Table S3: Global linkage disequilibrium tests for all locus pairs. Table S4: Pairwise Fst values corrected for null alleles using the ENA method in FreeNA. Table S5: Pairwise Rst values among the eight populations. Figure S1: Mantel test scatter plot.
