# Supplementary figures and images for: Genetic Diversity and Gene Flow of the Ectomycorrhizal Mushroom Lactarius hatsudake in Southern China: Evidence from SSR Markers
Source: J Fungi (Basel). 2026 Apr 15;12(4):280. doi: 10.3390/jof12040280 (PMC13117080; doi:10.3390/jof12040280)

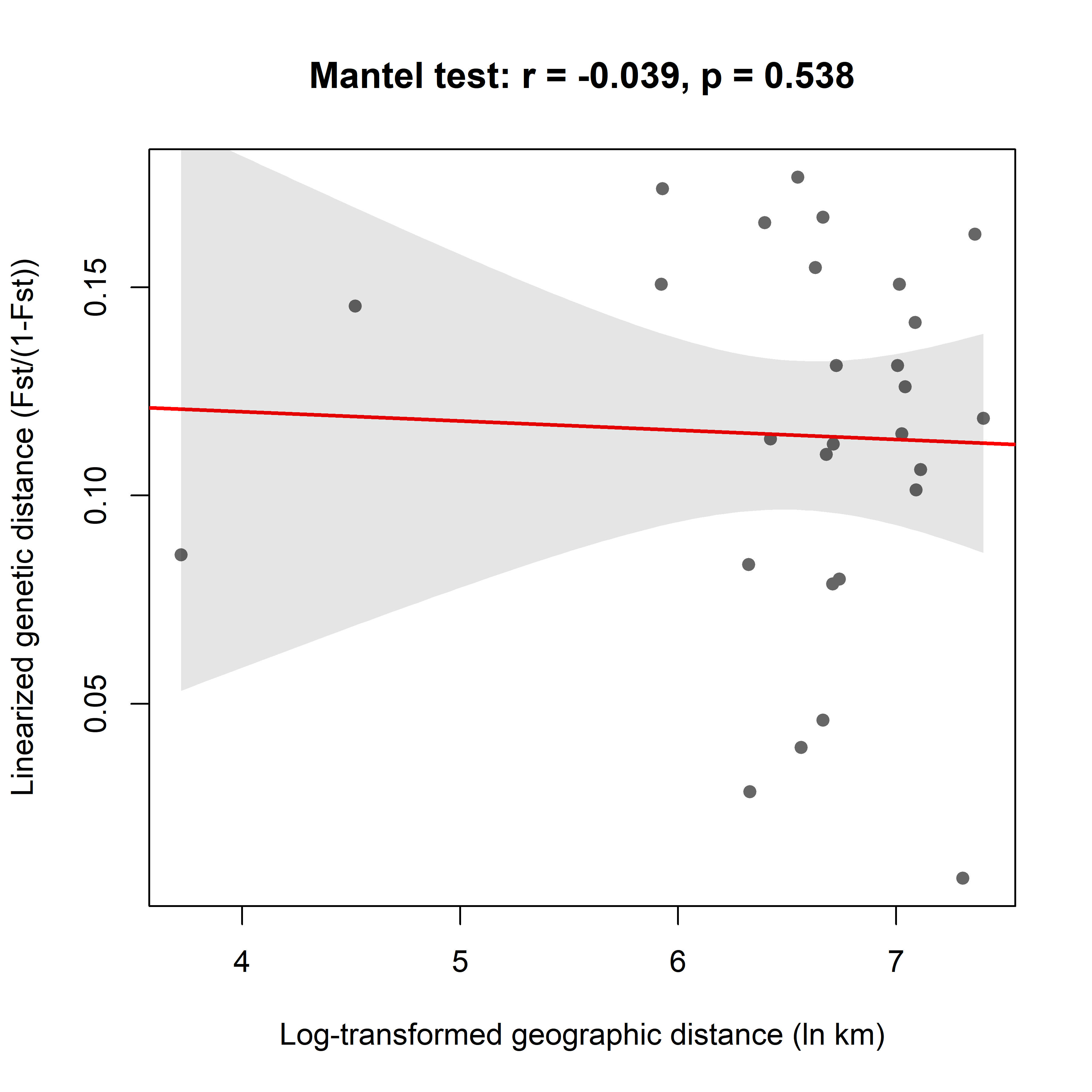

Supplement: Supplementary file 1 [file jof-12-00280-s001.zip › Figure S1.tif]
